# Zosteriform Dissemination of Molluscum Contagiosum Infection Diagnosed in an Immunocompetent Girl: A Rare Clinical Presentation

**DOI:** 10.1002/ccr3.70301

**Published:** 2025-03-07

**Authors:** Aya Marashli, Mohammad Adi, Zuheir Al‐shehabi, Lina Al‐Soufi

**Affiliations:** ^1^ Department of Dermatology National Hospital Latakia Syria; ^2^ Faculty of Medicine Damascus University Damascus Syria; ^3^ Faculty of Medicine Tishreen University Latakia Syria

**Keywords:** case report, herpes zoster‐like pattern, Molluscipoxvirus genus, molluscum contagiosum, molluscum contagiosum virus, zosteriform dissemination

## Abstract

Molluscum contagiosum (MC) is a benign, self‐limiting infectious lesion that primarily affects children and young adults, especially those with immunodeficiency. MC is caused by a poxvirus from the Molluscipoxvirus genus of the Poxviridae family. Diagnosis is mainly clinical, with lesions presenting as dome‐shaped, spherical, solid, flesh‐colored, pearly papules with central umbilication. MC commonly appears on the limbs, face, trunk, and axillary regions. The differential diagnosis includes inflammatory, infectious, and neoplastic causes, varying by age and immunological condition. This case report presents a rare zosteriform distribution of MC infection in an immunocompetent girl, a presentation described only twice in medical literature. The unusual distribution resulted from scratching, a process known as the Koebner phenomenon (pseudo‐koebnerization).

**Summary**

- MC can present in a zosteriform pattern, resembling herpes zoster.
- This uncommon presentation in an immunocompetent patient highlights the importance of recognizing various clinical forms for accurate diagnosis and management.
- Early diagnosis and proper management can prevent unnecessary treatments and reduce the risk of viral spread through scratching (Koebner phenomenon).

## 1 Introduction

Molluscum contagiosum (MC) is a common, self‐limiting infectious dermatosis caused by the molluscum contagiosum virus (MCV), a double‐stranded DNA virus from the poxvirus family. MC is usually transmitted through direct skin contact or indirectly via contaminated objects. It predominantly affects children aged 2–5, immunocompromised patients, and sexually active young adults [1]. Diagnosis is typically based on clinical features, with lesions appearing as dome‐shaped, flesh‐colored, umbilicated papules, 1–5 mm in diameter. While often asymptomatic, lesions can be tender or pruritic. The most common locations include the extremities, trunk, limbs, axillary areas, anogenital area, abdomen, and inner thighs [1, 2]. Differential diagnoses include keratoacanthoma, lichen planus, epidermal cyst, pyoderma, verruca vulgaris, folliculitis, cutaneous fungal disorders, basal cell carcinoma, condyloma acuminatum, and varicella‐zoster [2]. This report documents an exceptionally rare clinical presentation of MC.

## 2 Case History/Examination

**FIGURE 1:**
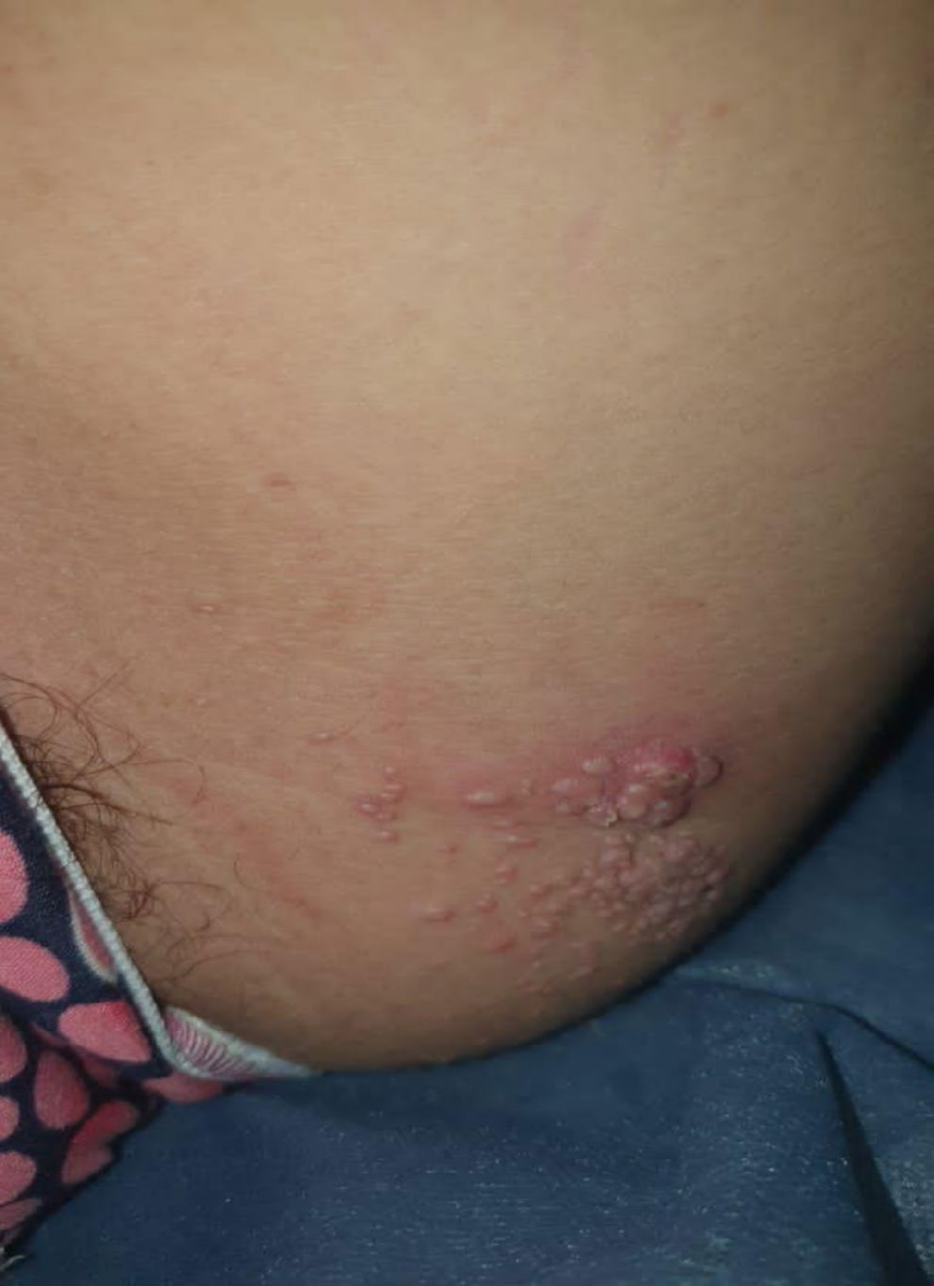
Small, reddish‐colored, pearly, dome‐shaped semi‐transparent papules measuring 2–4 mm in size, seen over the skin of the left thigh with a smooth and shiny appearance and grouped in clusters.

A 14‐year‐old girl presented to the dermatology department with mildly pruritic, flesh‐colored, umbilicated pearly papules. The lesions appeared in a zosteriform distribution on the inner left thigh and had been present for 3 months. The patient reported no pain or tingling sensations. The distribution of the lesions resembled a zoster virus infection (Figure 1) No other lesions were found elsewhere on the body. The patient was otherwise healthy, with no systemic disease or immunosuppression, and no history of surgery or medication was noted.

### 2.1 Differential Diagnosis

Two weeks before our consultation, the patient was misdiagnosed with impetigo and treated with Hexamidine antiseptic twice daily and Fusidic Acid three times daily for 7 days. The treatment caused the lesions to become more reddish and itchy. Subsequently, the patient developed a zosteriform distribution of molluscum contagiosum 10 days after initiating therapy. The number of molluscum contagiosum lesions began to increase from the fifth day of therapy and continued to rise until the tenth day, indicating a reaction likely triggered by the misdiagnosis and subsequent treatment. The diagnosis of impetigo was ruled out because of the absence of honey‐colored crusts, a hallmark of impetigo. In addition, we ruled out other differential diagnoses such as skin warts, folliculitis, and milia by the clinical appearance. Comprehensive STD screening, including tests for chlamydia, gonorrhea, and syphilis, was negative. Additionally, immunosuppression was assessed through HIV testing and a complete blood count (CBC), with all results indicating no underlying immune disorders. An examination of the skin revealed around 40 flesh‐colored, umbilicated papular lesions, each measuring 3–4 mm, on the left thigh, correlating with the clinical findings of MC infection. To confirm, dermatoscopy was performed, revealing characteristic features such as central umbilication, white to yellowish molluscum bodies, and crown vessels with a radial pattern. These findings reinforced the diagnosis of MC and helped differentiate it from similar conditions, including verruca vulgaris and folliculitis.

## 3 Conclusion and Results

**FIGURE 2:**
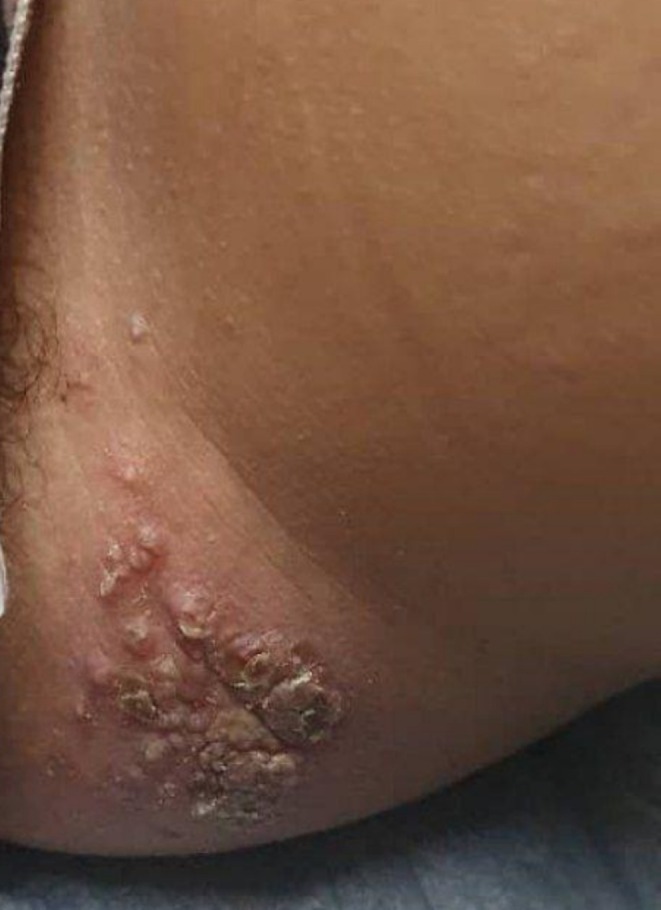
The patient at follow‐up after being treated with topical potassium hydroxide 10% for the MC lesions.

Based on the clinical examination and dermoscopic findings, we confirmed the diagnosis of MC and excluded the differential diagnoses. The patient applied a 10% potassium hydroxide (KOH) topical solution to the affected areas once daily before bedtime. The treatment was continued until the lesions were fully resolved. A positive response to treatment for molluscum contagiosum was observed, as illustrated in Figure 2. Improvement began on day 14 of the treatment, with lesions completely disappearing by day 45, without any complications or recurrence.

## 4 Discussion

MC accounts for about 1% of all dermatological diagnoses and is more frequent in regions with tropical and humid climates [3]. The standard lesion is solitary or multiple umbilicated dome‐shaped, spherical, and pinkish‐purple papules. These clinical features were present in our patient. Atypical manifestations may strongly resemble many cutaneous conditions, including warts, basal cell carcinoma, intradermal nevus, keratoacanthoma, and amelanotic melanoma, but it is extremely rare to mimic zoster virus infection dissemination [1, 4, 5]. Using PubMed, Scopus, and Google Scholar, we searched the following keywords: (Molluscum contagiosum) AND (zoster* OR Blaschko). The results showed that zosteriform‐like dissemination has been reported in two cases of MC in the medical literature. The first case involved an HIV‐positive female patient who presented with umbilicated pearly papules on the right side of her face for 2 months, successfully treated with curettage and 70% trichloroacetic acid. Zosteriform molluscum contagiosum developed after applying hydrocortisone 1% cream for eyelid dermatitis, likely due to the topical corticosteroid causing localized immune suppression [4, 6]. The second case was a 12‐year‐old girl who developed multiple umbilicated dome‐shaped papules in a zosteriform pattern on the right side of her trunk. This distribution was likely due to the use of desonide cream for atopic dermatitis, where the corticosteroid weakened the epidermal barrier, increasing vulnerability to viral infections. The Koebner phenomenon likely contributed to the linear spread of lesions following minor trauma or inflammation [5, 7]. As far as we know, our patient is the third reported case of zosteriform dissemination of MC infection and the second case in an immunocompetent patient worldwide. Scratching skin lesions facilitates the spread of viruses to other parts of the body and other people [3]. The molluscum contagiosum infection can be spread by scratching the lesions through a mechanism called pseudo‐koebnerization, a subtype of the Koebner phenomenon. This phenomenon seeds the infectious agents next to the infected area [7]. This mechanism is likely responsible for the zosteriform eruption of the lesions in our case. Molluscum contagiosum is frequent in the pediatric population and immunocompromised adults and is considered uncommon in the immunocompetent population [1]. The patient in our case was immunocompetent. MC lesions in children usually affect the trunk and the extremities, especially the intertriginous tissues, while inner thigh lesions are more common among adults in sexually transmitted forms [2].

In conclusion, our case suggests that dermatologists should consider various clinical features of MC to avoid misdiagnosis due to rare clinical distributions. Additionally, molluscum contagiosum should not be ruled out even in immunocompetent patients. Furthermore, it is essential to counsel patients against scratching the lesions to prevent the spread of (MCV).

## Author Contributions

**Aya Marashli:** data curation, investigation, methodology, writing – original draft, writing – review and editing. **Mohammad Adi:** writing – original draft, writing – review and editing. **Zuheir Al‐shehabi:** writing – original draft, writing – review and editing. **Lina Al‐Soufi:** project administration, resources, supervision, writing – review and editing.

## Consent

Written informed consent was obtained from the patient's parents to publish this report in accordance with the journal's patient consent policy.

## Conflicts of Interest

The authors declare no conflicts of interest.

## Data Availability

Datasets with DOIs are openly accessible in a public repository.
